# Discriminant analysis of occupational performance characteristics in patients with major depressive disorders and healthy individuals

**DOI:** 10.1002/pcn5.70038

**Published:** 2024-11-26

**Authors:** Tomonari Hayasaka, Izumi Nagashima, Miku Hoshino, Koji Teruya, Yasuyuki Matumoto, Masami Murao, Taku Maruki, Masako Watanabe, Takeshi Katagiri, Yayoi Imamura, Mariko Kurihara, Yuki Oe, Yoshikazu Takaesu, Takashi Tsuboi, Koichiro Watanabe, Hitoshi Sakurai

**Affiliations:** ^1^ Department of Rehabilitation Kyorin University Faculty of Health Sciences Tokyo Japan; ^2^ Department of Neuropsychiatry Kyorin University Faculty of Medicine Tokyo Japan; ^3^ Department of Neuropsychiatry Kyorin University Hospital Tokyo Japan; ^4^ Department of Health and Welfare Kyorin University Faculty of Health Sciences Tokyo Japan; ^5^ Department of Neuropsychiatry, Graduate School of Medicine University of the Ryukyus Okinawa Japan

**Keywords:** artistic activity, discrimination analysis, healthy, major depression, occupational therapy

## Abstract

**Aim:**

Assessing symptoms and daily functioning in patients with major depressive disorder (MDD) can be challenging, as their limited self‐monitoring abilities may result in behavior observed during structured interviews not accurately reflecting their daily lives. This study aimed to determine if specific occupational behaviors could distinguish individuals with MDD from healthy individuals.

**Methods:**

Baseline data were collected from medical records and activity programs. Three occupational therapists conducted content analysis to assess occupational performance characteristics. Chi‐squared tests compared the prevalence of these characteristics between patients with MDD and healthy controls. Multivariable logistic regression controlled for potential confounders, with independent variables selected based on clinical relevance and sample size (*p* < 0.01). Discriminant analysis was used to enhance group differentiation, assessing prediction rates using area under the curve (AUC) values.

**Results:**

A total of 69 occupational performance characteristics were identified, with 12 showing significant differences between 27 patients with MDD and 43 healthy controls. Key discriminators included “Ask questions and consult” (*p* < 0.001, odds ratio [OR] = 0.051, 95% confidence interval [CI] = 0.009–0.283), “Concentrate on work” (*p* = 0.003, OR = 0.078, 95% CI = 0.015–0.416), “Choose simple work” (*p* = 0.004, OR = 17.803, 95% CI = 2.446–129.597), and “Punctual” (*p* = 0.017, OR = 0.030, 95% CI = 0.002–0.530). Discriminant analysis using these variables yielded a Wilks' *λ* of 0.493 (*p* < 0.001), achieving an 88.6% accuracy rate. The receiver operating characteristic curve's AUC value was 0.911 (sensitivity = 95.3%, specificity = 77.8%).

**Conclusion:**

This study highlights the importance of occupational performance characteristics in tailoring treatment strategies for MDD, providing insights beyond traditional assessment methods.

## Graphical abstract

The present study revealed that four occupational performance characteristics were extracted to discriminate between depressed patients and normal subjects. The area under the curve values of the receiver operating characteristic curves generated from the discrimination scores indicated that their discriminative ability was of very high accuracy. Specific occupational performance characteristics are effective in distinguishing major depressive disorder (MDD) patients from healthy individuals and may serve as valuable indicators for screening high‐risk subjects in daily life. This novel insight, centered on behaviors not typically assessed through conventional methods, offers a new avenue for evaluating MDD. Looking ahead, there is potential to extend the application of occupational performance characteristics to other mental disorders beyond MDD.

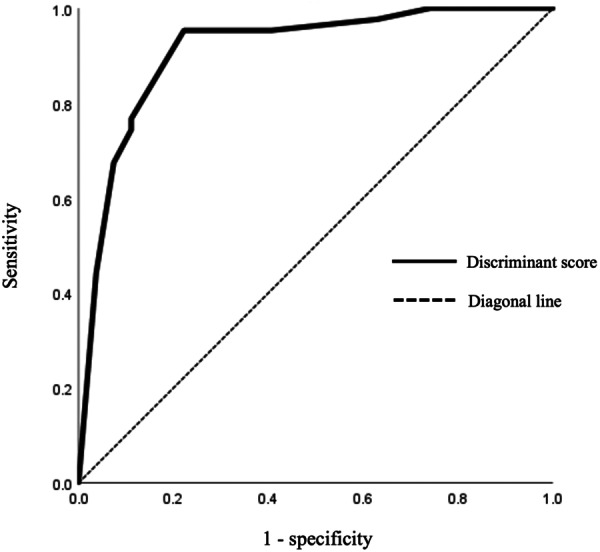

## INTRODUCTION

Major depressive disorder (MDD) is a common condition, affecting approximately 4.4% of the global population.^1^, ^2^ The primary symptoms of MDD include a persistent depressed mood and a loss of interest in previously enjoyed activities. These symptoms can result in a wide range of functional impairments, including compromised physical and cognitive functioning,^3^, ^4^ which hinder the ability to perform self‐care and daily activities. These impairments extend to academic and occupational settings, significantly affecting individuals' ability to attend school and maintain employment.^5^, ^6^, ^7^ As a result, patients with MDD often experience increased social isolation and altered or diminished roles within their families and society.^8^, ^9^ Such challenges substantially reduce their quality of life and lead to long‐term social and economic problems.^10^, ^11^

To assess the severity of MDD, clinicians commonly employ rating scales such as the Montgomery‐Åsberg Depression Rating Scale (MADRS) and the Hamilton Depression Rating Scale, both of which are considered reliable and valid tools based on clinical evaluations on patients' reports. Self‐administered rating scales, such as the Patient Health Questionnaire‐9 (PHQ‐9) and the Quick Inventory of Depressive Symptomatology, are also widely used to gauge depressive symptoms.^12^ However, the accuracy of these self‐reports can be compromised for several reasons.^13^ First, cognitive impairments are core clinical manifestations of MDD, including deficits in selective attention, working memory, and long‐term memory. These impairments are closely related to psychosocial dysfunction, tend to worsen with each depressive episode, and may persist even after other symptoms have remitted.^4^, ^14^ Second, patients with MDD may be reluctant to disclose information when answering questions about their daily life and symptoms. They may exaggerate improvements to present themselves favorably to clinicians or avoid discussing embarrassing or socially unacceptable behaviors.^13^ Furthermore, a patient's behavior during a clinical interview may not accurately reflect their behavior in daily life, as the clinical setting itself can be anxiety‐provoking, causing patients to behave in a more inhibited or guarded manner.^15^ In light of these challenges, objective assessment of behavior in situations similar to daily life may be more useful in diagnosing MDD.

Occupational therapy is a therapeutic intervention that assists individuals in developing, recovering, or maintaining daily living and work skills. It supports psychiatric rehabilitation through various interventions, including psychosocial, psychoeducational, cognitive, and exercise‐based approaches.^16^ The primary goal of occupational therapy is to help patients achieve independence and enhance their daily living skills through diverse activities.^17^, ^18^ This approach enables occupational therapists to assess daily living and social functions, thereby gaining insight into the impact of mental illness on a patient's social life.^19^ While most previous studies on MDD and occupational therapy have focused on the effects of interventions on psychosomatic symptoms and stress coping,^20^, ^21^ there is a notable lack of evidence regarding the impact of occupational therapy perspectives on the differential diagnosis of MDD.^22^

To address this issue, we conducted a retrospective survey comparing performance characteristics in occupational therapy between patients with MDD and healthy subjects. Our aim was to investigate whether specific occupational performance characteristics can effectively distinguish MDD patients from healthy individuals.

## METHODS

### Study design

This retrospective study analyzed data from patients who received a detailed assessment for difficult‐to‐treat depression at Kyorin University Hospital, Tokyo, Japan, from January 2015 to March 2019. In our comprehensive evaluation of difficult‐to‐treat depression, occupational therapy records provide valuable insights for examining depression in detail. As a preliminary step, we opted for conducting a retrospective study using medical records to generalize the detailed occupational therapy records for a more thorough examination of depression. All patients with difficult‐to‐treat depression were given the option to opt‐out. Healthy control participants were recruited from volunteers. All healthy control participants received a comprehensive explanation of the study, underwent the consent process, and provided written informed consent prior to their participation. Data from healthy control participants were recorded in a table, including both real‐time observations and video review ratings, which were subsequently analyzed.

### Participants

For the patient group, inclusion criteria were (a) a detailed assessment for difficult‐to‐treat depression conducted within the previous week, (b) an age range of 20–70 years, and (c) a diagnosis of MDD according to the Diagnostic and Statistical Manual of Mental Disorders, Fifth Edition (DSM‐5). Patients were excluded if they had (a) ongoing alcohol or substance abuse, (b) an emergent suicidal risk, (c) a diagnosis other than MDD according to the DSM‐5, or (d) severe physical illness. Healthy control participants were included based on the following criteria: (a) no history of mental illness, (b) age between 20 and 70 years, and (c) the PHQ‐9 scores below the designated cutoff.

### Occupational therapy program description

Occupational performance characteristics were evaluated during a single session of an artistic activity program in occupational therapy, with all participants attending only once. The program was divided into six stages, with occupational performance characteristics meticulously recorded at each stage (Table 1). To ensure consistency and avoid differential effects between the two groups, the introduction, conduct, and recording of the sessions were standardized. During the pre‐session instructions, each participant was individually interviewed and informed that the task was intended solely for behavioral assessment, not for therapeutic purposes. The program, conducted in group sessions with approximately 10 participants and facilitated by two occupational therapists, primarily involved creating artwork within 1 h. Various therapeutic activities were prepared, including coloring, origami, knitting, and leatherwork, offering participants a selection of over 10 tasks of varying difficulty levels. Additionally, participants documented their self‐evaluation status, mood status, and pre‐ and post‐program impressions on a scale from 1 (very poor) to 5 (excellent). These activities mimic daily living tasks, providing a more accurate assessment of behaviors than laboratory settings.^17^, ^18^, ^19^ The sessions, which maintained identical settings and activities, were conducted separately for patients with MDD and healthy controls.

**Table 1:** Implementation of artistic activity program.

Procedure	Details
(a) Start of the program	Promotion of motivation for participation
	Confirmation of participation
(c) Description of the program	Confirmation of content and purpose of the session
(d) Icebreaker	Self‐introduction of each participant
(e) Selection of artwork task	Free selection of task from more than 10 choices with different levels
(f) Implementation of the artwork task	Preparation of tools and materials
	Creation of selected artwork task
(h) End of the program	Present impressions for the session
	Cleaning up

### Occupational performance characteristics evaluation

Occupational therapists' observations focused on participants' abilities in communication, task engagement, and environmental adaptation. Following each session, occupational therapists evaluated and recorded the occupational performance characteristics of participants in their records. The behavioral assessments in occupational therapy were independently recorded by each occupational therapist. These records were categorized by three occupational therapists through a content analysis process. This process involved (1) extracting occupational performance characteristic‐related information from records, (2) removing unnecessary content to create raw data, (3) substantially condensing this data, (4) organizing it based on semantic similarities and converting it into equivalent but unaltered expressions, and (5) categorizing these into occupational performance characteristics. The classification of performance characteristics was then conducted collaboratively in the same room by the three therapists. In cases of disagreement, they discussed their perspectives and reached a consensus on the most appropriate classification.

### Statistical analysis

Baseline demographic and clinical data, including age, gender, educational background, marital status, and employment status, were extracted from records and program records of the artistic activity. The MADRS score and disease duration were collated for patients with MDD, and the PHQ‐9 score was collected for healthy controls. To compare the MDD patient group with the healthy control group across these variables, the Mann–Whitney *U*‐test and either the *χ* ^2^ test or Fisher's exact test were utilized. The occurrence of MDD patients and healthy controls within each occupational performance characteristic category was compared using the *χ* ^2^ test. Multivariable logistic regression was employed to analyze occupational performance characteristics for both MDD patients and healthy control participants, controlling for potential confounding variables. Age and gender, which may influence the dependent variable, were included as adjustment variables. Participants' ages were recorded at the time of the behavioral assessment and incorporated as a continuous variable. Gender was coded as a dummy variable, with male as the reference Group (0) and female as the comparison Group (1). The analysis distinguished between the MDD patient and healthy control groups as the dependent variable. Independent variables included those occupational performance characteristics identified as significant (*p* < 0.01) in the chi‐square test, selected based on clinical significance and sample size. Multicollinearity was checked using the variance inflation factor (VIF). Discriminant analysis, using the stepwise method, was conducted to further differentiate between the MDD patient and healthy control groups, with the same dependent variable and occupational performance characteristics as independent variables. Receiver operating characteristic (ROC) curves were then constructed to assess the precision of these evaluations in distinguishing between the two groups. The prediction rates obtained using area under the curve (AUC) values were considered high if the accuracy was >0.9, moderate if between 0.7 and 0.9, and low if between 0.5 and 0.7.^23^ All statistical analyses were carried out using SPSS version 28 software for Windows (SPSS Inc.). A P‐value of <0.05 was deemed to indicate statistical significance.

## RESULTS

A total of 27 MDD patients (13 males, average age 44.3 ± 9.8 years) and 43 healthy control participants (22 males, average age 42.3 ± 7.3 years) met the criteria. Baseline demographic and clinical variable comparisons between the MDD patient group and the healthy control group revealed significant differences in educational background (P < 0.001) and employment status (*P* < 0.001), with the healthy control group demonstrating higher rates in both categories (Table 2). No significant differences were found in other variables. The MADRS subitems of the MDD patients were as follows: apparent sadness 1.9 ± 1.6, reported sadness 2.1 ± 2.1, inner tension 1.4 ± 1.3, reduced sleep 1.4 ± 1.5, reduced appetite 1.0 ± 1.4, concentration loss 2.0 ± 1.4, lassitude 1.8 ± 1.7, inability to feel guilty 1.8 ± 1.7, pessimistic thoughts 2.0 ± 1.5, and suicidal thoughts 1.7 ± 1.7.

**Table 2:** Comparison of demographic and clinical valuables between the MDD patient group and the healthy control group.

Variables	Mean ± SD or *n* (%)		I‐value
	MDD patient group (*n* = 27)	Healthy control group (*n* = 43)	
Age (years)	43.0 ± 12.4	41.1 ± 8.3	0.510
Gender (male)	13 (48.1%)	22 (51.2%)	0.806
Marital status (spouse)	15 (55.6%)	31 (73.8%)	0.156
Educational background (university degree)	15 (55.6%)	40 (93.0%)	<0.001*
Employment (doing)	3 (11.1%)	41 (95.3%)	<0.001*
Disease duration (years)	9.1 ± 7.9	n/a	n/a
MADRS scores (points)	16.8 ± 9.7	n/a	n/a
PHQ‐9 scores (points)	n/a	1.1 ± 1.2	n/a
Applicable occupational performance characteristic (numbers)	9.4 ± 2.2	10.0 ± 3.7	0.423

Abbreviations: MADRS, Montgomery Åsberg Depression Rating Scale; n/a, not applicable; PHQ‐9, Patient Health. Questionnaire‐9; SD, standard deviation.

* *p* < 0.001.

A total of 69 occupational performance characteristics were identified from both groups (Table 1). Among these, 12 characteristics showed significant differences between the groups (Table 3). Multivariate logistic regression analysis indicated several factors significantly distinguishing the MDD patient group from the healthy control group. These factors included “Ask questions and consult” (*p* < 0.001, odds ratio [OR] = 0.051, 95% confidence interval [CI] = 0.009–0.283), “Concentrate on work” (*p* = 0.003, OR = 0.078, 95% CI = 0.015–0.416), “Choose simple work” (*p* = 0.004, OR = 17.803, 95% CI = 2.446–129.597), and “Punctual” (*p* = 0.017, OR = 0.030, 95% CI = 0.002–0.530). The healthy control group had more individuals who “Ask questions and consult,” “Concentrate on work,” and are “Punctual,” whereas the MDD patient group had more individuals who “Choose simple work.” After adjusting for age and gender, which were identified as confounding variables, the strong association between depression and specific work performance characteristics remained statistically significant (*p* < 0.05). None of the VIF values exceeded 10, and the mean VIF of the model was <6, indicating no collinearity in the model.

**Table 3:** Comparison of occupational performance characteristics between the MDD patient group and the healthy control group.

Variables	*n* (%)		*P*‐value
	MDD patient group (*n* = 27)	Healthy control group (*n* = 43)	
Punctual	18 (66.7%)	42 (97.7%)	<0.001***
Ask questions and consult	8 (29.6%)	33 (76.7%)	<0.001***
Lapses in the process (e.g., OT notes incomplete)	3 (11.1%)	21 (48.8%)	<0.001***
Concentrate on work	10 (37.0%)	32 (74.4%)	0.002**
State that OT is fun	9 (33.3%)	29 (67.4%)	0.005**
Choose simple work	10 (37.0%)	4 (9.3%)	0.006**
Never or rarely conversing with others	11 (40.7%)	6 (14.0%)	0.013*
Frequent changes in emotions during activities	4 (14.8%)	0 (0.0%)	0.019*
Feel uncomfortable in groups	4 (14.8%)	0 (0.0%)	0.019*
Calm as you get used to the place	10 (37.0%)	6 (14.0%)	0.027*
Expressionless	5 (18.5%)	1 (2.3%)	0.029*
Refuse suggestions	6 (22.2%)	2 (4.7%)	0.033*

Abbreviations: MDD, major depressive disorder; OT, occupational therapy.

* *P* < 0.05

** *P* < 0.01

*** *P* < 0.001.

**Figure 1:**
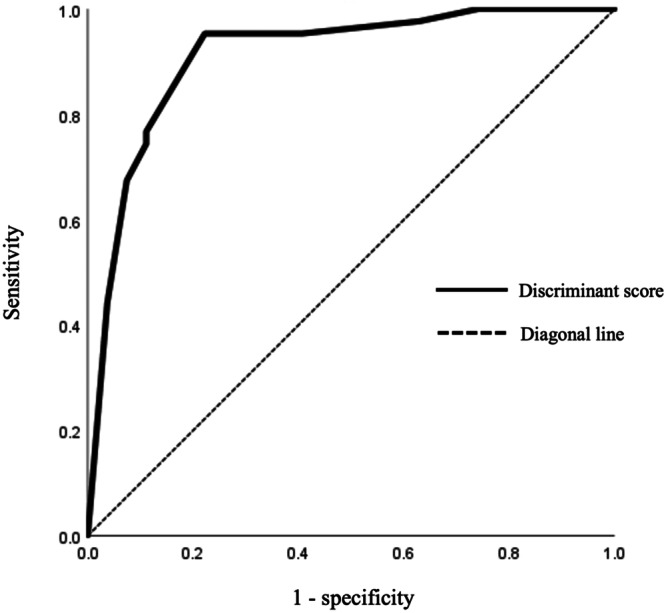
Receiver operating characteristic curves of discriminant score.

Discriminant analysis was conducted using the distinction between the MDD patient group and the healthy control group as the dependent variable, and the aforementioned four variables as independent variables. To ensure the ROC curve was consistently oriented, the score for “Choose simple work” was reversed, giving all data positive discriminant scores. The results indicated that these variables produced a linear discriminant formula: 1.485 × “Ask questions and consult” + 1.475 × “Punctual” + 1.430 × “Choose simple work (*reversal item)” + 1.332 × “Concentrate on work”−4.078. Wilks' *λ* was 0.493 (P < 0.001), and the true discrimination rate was 88.6% (Table 4). When the ROC curve for discriminating the MDD patient group from the healthy control group was constructed using the discriminant scores obtained from the linear discriminant formula, the AUC was 0.911 (95% CI = 0.835–0.986, *P* < 0.001), indicating very high accuracy, with sensitivity and specificity of 95.3% and 77.8%, respectively, at a cutoff value of—0.479 (Figure 1).

**Table 4:** Discriminant analysis by occupational performance characteristics.

Analytic factors	Standardized canonical discriminant function coefficient	Canonical discriminant function coefficient	*P*‐value
Ask questions and consult	0.657	1.485	<0.001***
Concentrate on work	0.615	1.332	0.002**
Choose simple work (*reversal item)	0.546	1.430	0.004**
Punctual	0.472	1.475	<0.001***

*Note*: These variables produced a linear discriminant formula: 1.485 × “Ask questions and consult” + 1.475 × “Punctual” + 1.430 × “Choose simple work (*reversal item)” + 1.332 × “Concentrate on work”−4.078.

*P < 0.05

** *P* < 0.01

*** *P* < 0.001.

## DISCUSSION

To the best of our knowledge, this is the first retrospective study to examine the behavioral characteristics of MDD patients from an occupational therapy perspective and compare them with those of healthy subjects. Multivariate logistic regression and discriminant analyses identified four occupational performance characteristics that effectively distinguished between the two groups: “Ask questions and consult,” “Punctual,” “Concentrate on work,” and “Choose simple work.” These four characteristics yielded a linear discriminant formula: 1.485 × “Ask questions and consult” + 1.475 × “Punctual” + 1.430 × “Choose simple work (*reversal item)” + 1.332 × “Concentrate on work” −4.078, which demonstrated a discrimination rate of 88.6%. These findings suggest that specific occupational performance characteristics are effective in distinguishing MDD patients from healthy individuals.

Each of the four occupational performance characteristics are consistent with known attributes of MDD. The characteristic “Ask questions and consult” indicates that MDD patients were less likely than healthy controls to seek help or ask questions about tasks they did not understand. This behavior is likely a result of the social isolation experienced by MDD patients, fostering a pessimistic view of their relationships with others and leading to hesitancy in interactions.^24^ Providing support for consultation within the workplace and community mental health settings may help mitigate the difficulty of MDD.^25^, ^26^, ^27^ For the characteristic “Punctual,” significant differences were noted in the ability to arrive at a predetermined location on time for scheduled work. MDD can impair time management abilities due to a decreased perception of time passage associated with the illness.^28^ Reduced motivation may also hinder the ability to engage in daily tasks, complicating the smooth execution of activities.^29^ Additionally, sleep disturbances, common in MDD, can negatively impact time management.^30^ In the MDD patient group, these symptoms likely contribute to difficulties in managing time effectively. Regarding “Concentrate on work,” significant differences were observed in the ability to focus on tasks and maintain stable work performance. The diagnostic criteria for MDD in the DSM include decreased concentration.^31^, ^32^ In this study, while the MADRS score for the MDD patient group was 16.8 ± 9.7, the item assessing decreased concentration was 2.0 ± 1.4 points. This symptom likely made it difficult for patients to sustain stable work performance.^33^, ^34^ For the characteristic “Choose simple work,” MDD patients were observed to select less difficult tasks, while healthy subjects tended to choose more challenging ones. This avoidant behavior is often due to low self‐esteem, with MDD patients aiming to temporarily alleviate negative emotions such as anxiety and sadness.^35^, ^36^ In general societal contexts, individuals with MDD may also choose simpler tasks to avoid high‐stress situations. These findings highlight specific occupational performance characteristics that can distinguish MDD patients from healthy individuals, offering valuable insights for screening and supporting high‐risk individuals in daily life.

The AUC value of the ROC curve based on the discriminant score was 0.911 (95% CI 0.835–0.986, *p* < 0.001), indicating a very high level of accuracy. Occupational therapy facilitates behavioral assessments in environments that closely simulate daily life, allowing for the identification of behavioral characteristics that may be overlooked in self‐administered surveys or structured interviews. By incorporating activities from a person's daily routine, occupational therapy replicates real‐life scenarios, offering a unique opportunity to assess social skills while simultaneously reducing anxiety and improving alertness. This therapeutic approach inherently fosters the reproduction of everyday behaviors and actions. In this study, it became evident that depression has a significant impact on daily functioning, and the occupational therapy assessment provided valuable insights for distinguishing between patients with depression and healthy controls. These findings may inform the development of new occupational therapy assessment tools to improve diagnostic accuracy.

There are several limitations to this study. First, the sample size was small and the study was conducted at a single university hospital, which limits the generalizability of the results. Future research should involve collaboration with multiple medical institutions offering occupational therapy programs and should utilize larger sample sizes to enhance the validity and applicability of the results. Second, the occupational performance characteristics were selected based on a retrospective survey, potentially introducing bias in the classification and quality of the information. Third, while the comparison of occupational performance characteristics between MDD patients and healthy subjects revealed significant differences in 12 items, the reason for the higher frequency of “Lapses in the process (e.g., incomplete OT notes)” in the healthy group compared to the MDD group was not fully explored (Table 3 and S1 have been revised). Fourth, although the occupational performance characteristics evaluated behavior during the artistic activity program in occupational therapy, they did not assess behavior in daily life. Fifth, aside from age and gender, which were considered confounding factors in this study, other variables such as work history and educational background may also impact behavioral characteristics. These factors should be explored in future research to further clarify their influence. Finally, the relationships between the various occupational performance characteristics could not be fully examined.

In conclusion, our study identified four occupational performance characteristics that effectively discriminate between depressed patients and healthy individuals. The AUC values of the ROC curves generated from the discrimination scores demonstrated a very high level of accuracy in their discriminative ability. These findings highlight specific occupational performance characteristics that can differentiate patients with MDD from healthy individuals, providing valuable information for screening and supporting at‐risk individuals in their daily lives. The identified occupational performance characteristics could be practically implemented in clinical settings to assess individuals at high risk for depression by monitoring specific aspects of their daily activities. Additionally, incorporating these characteristics into checklists used in daily life support for patients with depression may help prevent recurrence by encouraging regular review and adjustment of their routines. This novel insight, focused on behaviors not typically assessed through conventional methods, presents a new avenue for evaluating MDD. Looking ahead, there is significant potential to extend the application of occupational performance characteristics to other mental disorders beyond MDD.

## AUTHOR CONTRIBUTIONS

**Tomonari Hayasaka**: Acquisition and analysis of data, and drafting the manuscript. **Izumi Nagashima**: Acquisition and analysis of data, and revising the manuscript. **Miku Hoshino**: Acquisition and analysis of data. **Koji Teruya**: Acquisition and analysis of data. **Yasuyuki Matumoto**: Acquisition and analysis of data. **Masami Murao**: Acquisition and analysis of data. **Taku Maruki**: Acquisition and analysis of data. **Masako Watanabe**: Acquisition and analysis of data. **Takeshi Katagiri**: Acquisition and analysis of data. **Yayoi Imamura**: Acquisition and analysis of data. **Mariko Kurihara**: Acquisition and analysis of data. **Yuki Oe**: Acquisition and analysis of data. **Yoshikazu Takaesu**: Acquisition and analysis of data. **Takashi Tsuboi**: Acquisition and analysis of data. **Koichiro Watanabe**: Acquisition and analysis of data. **Hitoshi Sakurai**: Conception and design of the study, and acquisition and analysis of data.

## CONFLICT OF INTEREST STATEMENT

Dr. Tomonari Hayasaka, Dr. Izumi Nagashima, Ms. Miku Hoshino, Dr. Koji Teruya, Dr. Taku Maruki, Dr. Masako Watanabe, Dr. Takeshi Katagiri, Dr. Yayoi Imamura, Ms. Mariko Kurihara, and Mr. Yuki Oe have nothing to declare. Dr. Masami Murao received honorariums from Sumitomo Pharma and Yoshitomiyakuhin. Dr. Yasuyuki Matumoto received grants from the Japan Society for the Promotion of Science and honorariums from Sumitomo Pharma, Janssen Pharmaceutical, and Meiji Seika Pharma. Dr. Takaesu has received lecture fees from Takeda Pharmaceutical, Sumitomo Dainippon Pharma, Otsuka Pharmaceutical, Meiji Seika Pharma, Kyowa Pharmaceutical, Eisai, MSD, Yoshitomi, and research funding from Otsuka Pharmaceutical, Meiji Seika Pharma, MSD, and Eisai. Dr. Takashi Tsuboi received grants from the Japan Society for the Promotion of Science and honorariums from Takeda Pharmaceutical, Otsuka Pharmaceutical, Meiji Seika Pharma, Shionogi Pharma, Yoshitomiyakuhin, Sumitomo Pharma, Kyowa Pharmaceutical, MSD, Nippon Boehringer lngelheim, Mylan EPD, Mitsubishi Tanabe Pharma, Viatris, Mochida Pharmaceutical, Janssen Pharmaceutical, TEIJIN PHARMA, and Lundbeck Japan. Dr. Koichiro Watanabe is a consultant of Boehringer Ingelheim, Daiichi Sankyo, Eisai, Eli Lily, Janssen Pharmaceutical, Kyowa Pharmaceutical, Lundbeck Japan, Luye Pharma, Mitsubishi Tanabe Pharma, Otsuka Pharmaceutical, Lundbeck Japan, Luye Pharma, Mitsubishi Tanabe Pharma, Otsuka Pharmaceutical, Pfizer, Sumitomo Dainippon Pharma, Taisho Pharmaceutical, and Takeda Pharmaceutical. Dr. Hitoshi Sakurai received grants from the Japan Society for the Promotion of Science, Japan Research Foundation Clinical Pharmacology, and Takeda Science Foundation, and honorariums from Eisai, Takeda Pharmaceutical, Otsuka Pharmaceutical, Meiji Seika Pharma, Shionogi Pharma, Yoshitomiyakuhin, Sumitomo Pharma, Kyowa Pharmaceutical, MSD, Viatris, and Lundbeck Japan.

## ETHICS APPROVAL STATEMENT

This study was approved by the institutional review board of School of Medicine, Kyorin University (R03‐225).

## PATIENT CONSENT STATEMENT

Participants diagnosed with MDD were given the option to opt‐out. All healthy control participants signed an informed consent statement before participating in the study.

## CLINICAL TRIAL REGISTRATION

N/A.

## Supporting information

Supplementary Table 1.

## Data Availability

The data that support the findings of this study are openly available in figshare at https://doi.org/10.6084/m9.figshare.26172316.

## References

[1] Friedrich MJ . Depression is the leading cause of disability around the world. JAMA. 2017;317:1517. 10.1001/jama.2017.3826 28418490

[2] Baldessarini RJ , Forte A , Selle V , Sim K , Tondo L , Undurraga J , et al. Morbidity in depressive disorders. Psychother Psychosom. 2017;86:65–72. 28183075 10.1159/000448661

[3] Bair MJ , Robinson RL , Katon W , Kroenke K . Depression and pain comorbidity: a literature review. Arch Intern Med. 2003;163:2433. 14609780 10.1001/archinte.163.20.2433

[4] Porter RJ , Douglas KM . Cognitive impairment in people remitted from major depression. Lancet Psychiatry. 2019;6:799–800. 31422921 10.1016/S2215-0366(19)30278-0

[5] Sivertsen H , Bjørkløf GH , Engedal K , Selbæk G , Helvik A‐S . Depression and quality of life in older persons: a review. Dementia Geriatr Cognit Disord. 2015;40:311–339. 10.1159/000437299 26360014

[6] Kolovos S , Kleiboer A , Cuijpers P . Effect of psychotherapy for depression on quality of life: meta‐analysis. Br J Psychiatry. 2016;209:460–468. 27539296 10.1192/bjp.bp.115.175059

[7] Boreham ID , Schutte NS . The relationship between purpose in life and depression and anxiety: A meta‐analysis. J Clin Psychol. 2023;79:2736–2767. 37572371 10.1002/jclp.23576

[8] Courtin E , Knapp M . Social isolation, loneliness and health in old age: a scoping review. Health Soc Care Community. 2017;25:799–812. 26712585 10.1111/hsc.12311

[9] Vrshek‐Schallhorn S , Stroud CB , Mineka S , Hammen C , Zinbarg RE , Wolitzky‐Taylor K , et al. Chronic and episodic interpersonal stress as statistically unique predictors of depression in two samples of emerging adults. J Abnorm Psychol. 2015;124:918–932. 26301973 10.1037/abn0000088 PMC4948584

[10] Werner‐Seidler A , Perry Y , Calear AL , Newby JM , Christensen H . School‐based depression and anxiety prevention programs for young people: a systematic review and meta‐analysis. Clin Psychol Rev. 2017;51:30–47. 27821267 10.1016/j.cpr.2016.10.005

[11] Carolan S , Harris PR , Cavanagh K . Improving employee well‐being and effectiveness: systematic review and meta‐analysis of web‐based psychological interventions delivered in the workplace. J Med Internet Res. 2017;19:e271. 28747293 10.2196/jmir.7583 PMC5550734

[12] Furukawa TA . Assessment of mood: guides for clinicians. J Psychosom Res. 2010;68:581–589. 20488276 10.1016/j.jpsychores.2009.05.003

[13] Sawada N , Uchida H , Watanabe K , Kikuchi T , Suzuki T , Kashima H , et al. How successful are physicians in eliciting the truth from their patients?: A large‐scale internet survey from patients' perspectives [CME]. J Clin Psychiatry. 2012;73:311–317. 22490259 10.4088/JCP.11m07078

[14] Semkovska M , Quinlivan L , O'Grady T , Johnson R , Collins A , O'Connor J , et al. Cognitive function following a major depressive episode: a systematic review and meta‐analysis. Lancet Psychiatry. 2019;6:851–861. 31422920 10.1016/S2215-0366(19)30291-3

[15] Stockings E , Degenhardt L , Lee YY , Mihalopoulos C , Liu A , Hobbs M , et al. Symptom screening scales for detecting major depressive disorder in children and adolescents: a systematic review and meta‐analysis of reliability, validity and diagnostic utility. J Affect Disord. 2015;174:447–463. 25553406 10.1016/j.jad.2014.11.061

[16] Rocamora‐Montenegro M , Compañ‐Gabucio L‐M , Garcia De La Hera M . Occupational therapy interventions for adults with severe mental illness: a scoping review. BMJ Open. 2021;11:e047467. 10.1136/bmjopen-2020-047467 PMC8559113 34716157

[17] Arbesman M , Logsdon DW . Occupational therapy interventions for employment and education for adults with serious mental illness: a systematic review. Am J Occup Ther. 2011;65:238–246. 21675329 10.5014/ajot.2011.001289

[18] Gibson RW , D'Amico M , Jaffe L , Arbesman M . Occupational therapy interventions for recovery in the areas of community integration and normative life roles for adults with serious mental illness: a systematic review. Am J Occup Ther. 2011;65:247–256. 21675330 10.5014/ajot.2011.001297

[19] Viertiö S , Tuulio‐Henriksson A , Perälä J , Saarni SI , Koskinen S , Sihvonen M , et al. Activities of daily living, social functioning and their determinants in persons with psychotic disorder. Eur Psychiatry. 2012;27:409–415. 21377336 10.1016/j.eurpsy.2010.12.005

[20] Ramano EM , De Beer M , Roos JL . The perceptions of adult psychiatric inpatients with major depressive disorder towards occupational therapy activity‐based groups. S Afr J Psychiatr. 2021;27:a1612. 10.4102/sajpsychiatry.v27i0.1612 PMC8008034 33824758

[21] Hees HL , Koeter MW , De Vries G , Ooteman W , Schene AH . Effectiveness of adjuvant occupational therapy in employees with depression: design of a randomized controlled trial. BMC Public Health. 2010;10:558. 20849619 10.1186/1471-2458-10-558 PMC2946299

[22] Farah WH , Alsawas M , Mainou M , Alahdab F , Farah MH , Ahmed AT , et al. Non‐pharmacological treatment of depression: a systematic review and evidence map. Evid Based Med. 2016;21:214–221. 27836921 10.1136/ebmed-2016-110522

[23] Swets JA . Measuring the accuracy of diagnostic systems. Science. 1988;240:1285–1293. 3287615 10.1126/science.3287615

[24] Felsten G . Gender and coping: use of distinct strategies and associations with stress and depression. Anxiety Stress Coping. 1998;11:289–309.

[25] Castle NG , Engberg J . Organizational characteristics associated with staff turnover in nursing homes. Gerontologist. 2006;46:62–73. 16452285 10.1093/geront/46.1.62

[26] Suzumura M , FUSHIKI Y , KOBAYASHI K , OURA A , SUZUMURA S , YAMASHITA M , et al. A cross‐sectional study on association of work environment, coping style, and other risk factors with depression among caregivers in group homes in Japan. Ind Health. 2013;51:417–423. 23648771 10.2486/indhealth.2012-0204

[27] Gillies D , Buykx P , Parker AG , Hetrick SE . Consultation liaison in primary care for people with mental disorders. Cochrane Database Syst Rev. 2015;2015:CD007193. 26384252 10.1002/14651858.CD007193.pub2 PMC6463953

[28] Rosenheck R . Intense case management for severe mental health problems reduces time in hospital and loss to follow‐up compared with standard care, but benefits over non‐ICM are less clear. Evid Based Ment Health. 2011;14:2929. 10.1136/ebmh.14.1.29 21266626

[29] Wang P , Wang X . Effect of time management training on anxiety, depression, and sleep quality. Iran J Public Health. 2018;47:1822. 30788296 PMC6379615

[30] Miguel AQC , Tempski P , Kobayasi R , Mayer FB , Martins MA . Predictive factors of quality of life among medical students: results from a multicentric study. BMC Psychol. 2021;9:36. 33632321 10.1186/s40359-021-00534-5 PMC7905855

[31] Uher R , Payne JL , Pavlova B , Perlis RH . Major Depressive Disorder in DSM‐5: implications for clinical practice and research of changes from DSM‐IV: Review: Major Depressive Disorder in DSM‐5. Depress Anxiety. 2014;31:459–471. 24272961 10.1002/da.22217

[32] Kendler KS . The phenomenology of major depression and the representativeness and nature of DSM criteria. Am J Psychiatry. 2016;173:771–780. 27138588 10.1176/appi.ajp.2016.15121509

[33] Müller M . Differentiating moderate and severe depression using the Montgomery–Åsberg depression rating scale (MADRS). J Affect Disord. 2003;77:255–260. 14612225 10.1016/s0165-0327(02)00120-9

[34] Fried EI , Epskamp S , Nesse RM , Tuerlinckx F , Borsboom D . What are “good” depression symptoms? Comparing the centrality of DSM and non‐DSM symptoms of depression in a network analysis. J Affect Disord. 2016;189:314–320. 26458184 10.1016/j.jad.2015.09.005

[35] Felsten G . Minor stressors and depressed mood: reactivity is more strongly correlated than total stress. Stress Health. 2002;18:75–81.

[36] Haskell AM , Britton PC , Servatius RJ . Toward an assessment of escape/avoidance coping in depression. Behav Brain Res. 2020;381:112363. 31739002 10.1016/j.bbr.2019.112363

