# Supplementary material for: Discriminant analysis of occupational performance characteristics in patients with major depressive disorders and healthy individuals
Source: PCN Rep. 2024 Nov 26;3(4):e70038. doi: 10.1002/pcn5.70038 (PMC11598739; doi:10.1002/pcn5.70038)
Supplement: Supplementary file 1 — Supplementary Table 1. [file PCN5-3-e70038-s001.docx]

Supplementary Table 1. Occupational performance characteristics evaluated through an artistic activity program in occupational therapy

| **No.** | **OPC (No.1-18)** | **No.** | **OPC (No.19-36)** | **No.** | **OPC (No.37-54)** | **No.** | **OPC (No.55-69)** |
| --- | --- | --- | --- | --- | --- | --- | --- |
| 1 | Accept suggestions from others | 19 | Discard the work without any attachment | 37 | Makeshift work | 55 | Soliloquize while working |
| 2 | Altruism | 20 | Dislike (or state dislike for) artistic activities | 38 | Messy (i.e. difficulty in organizing things) | 56 | Start to talk to others once adapted |
| 3 | Ask questions and consult | 21 | Excessive confirmation | 39 | Messy work | 57 | State that OT is fun |
| 4 | Bring personal items into the OT room | 22 | Excessive reactions to others | 40 | Misunderstand processes | 58 | Sudden interruption of work |
| 5 | Calm as you get used to the place | 23 | Expressionless | 41 | Nervous in interpersonal interactions | 59 | Superficial communication |
| 6 | Careful and attentive in working | 24 | Feel down and decrease self-esteem | 42 | Never or rarely converse with others | 60 | Take a break during the program |
| 7 | Change activities frequently | 25 | Feel uncomfortable in groups | 43 | No eye contact | 61 | Take a long time to be seated |
| 8 | Choose or seek new activities | 26 | Fluent conversations | 44 | Not seek help | 62 | Try to get others to do their work for them |
| 9 | Choose simple work | 27 | Frequent changes in emotions during activities | 45 | Obvious fatigue | 63 | Unclear conversation contents |
| 10 | Clean up or help clean up | 28 | Give instructions to medical staff during activities | 46 | Pleased to be praised | 64 | Unstable work |
| 11 | Clearly uplifting mood | 29 | Have its own manners | 47 | Punctual | 65 | Untidy at working area |
| 12 | Concentrate on work | 30 | Have negative remarks (e.g. "I can't.") | 48 | Refuse suggestions | 66 | Use multiple colors when creating artworks |
| 13 | Create work at a high degree of perfection | 31 | High self-esteem | 49 | Repetition of the same behavior | 67 | Work on your own without using a textbook |
| 14 | Difficulty in choosing activities | 32 | Impaired body schema | 50 | Restless | 68 | Work only at your own pace |
| 15 | Difficulty in continuously participating in OT | 33 | Lack of completion | 51 | Roundabout way of conversation | 69 | Work too far ahead |
| 16 | Difficulty in finishing work | 34 | Lack of manual dexterity | 52 | Satisfaction with OT |  |  |
| 17 | Difficulty in handling some tools | 35 | Lapses in the process (ex; OT notes incomplete) | 53 | Self-rating 2 levels up after OT |  |  |
| 18 | Difficulty in understanding procedures | 36 | Lots of comments in OT note | 54 | Shy during conversations |  |  |

OPC, occupational performance characteristic; OT, occupational therapy

The order of listing is A to Z.
